# A study in Bangladesh, Colombia, and Uganda on creating and retaining mobile health survey panels for longitudinal data collection

**DOI:** 10.1038/s41598-025-16809-5

**Published:** 2025-09-25

**Authors:** Gulam Muhammed Al Kibria, Saifuddin Ahmed, Michelle R. Kaufman, Elizeus Rutebemberwa, Iqbal Ansary Khan, Tahmina Shirin, Andres Vecino Ortiz, Joseph Ali, Julian Fernandez Nino, Fernando Ruiz-Vallejo, Carolina Saavedra, Sandra Agudelo-Londoño, Dustin G. Gibson

**Affiliations:** 1 Department of International Health, Johns Hopkins University Bloomberg School of Public Health, 615 N Wolfe Street W5501, Baltimore, MD 21205 USA; 2 Department of Population, Family and Reproductive Health, Johns Hopkins University Bloomberg School of Public Health, Baltimore, MD 21205 USA; 3 Department of Health, Behavior and Society, Johns Hopkins University Bloomberg School of Public Health, Baltimore, MD 21205 USA; 4 Department of Health Policy, Planning and Management, School of Public Health, Makerere University, Kampala, Uganda; 5 Institute of Epidemiology, Disease Control and Research, Dhaka, Bangladesh; 6 Pontificia Universidad Javeriana, Bogotá, Colombia

**Keywords:** Mobile phone survey, Survey panel, Interactive voice response survey, Medical research, Epidemiology, Risk factors

## Abstract

The increased subscription and ownership of mobile phones have created opportunities to improve health, education, or economic outcomes, including mobile phone surveys (MPS) to collect health data. Most MPS used cross-sectional survey designs. We explored the potential of MPS to collect panel data using anonymous surveys with agreement in age and gender, and participants’ retention across survey waves in three low- and middle-income countries (LMICs): Bangladesh, Colombia, and Uganda. Using random digit dialing, participants were recruited from 6 age-gender strata (i.e., 18-29-, 30-44-, and 45+-year-old males and females). Three interactive voice response survey waves were sent at two-week intervals. In Wave 1, the number of complete interviews in Bangladesh, Colombia, and Uganda was 2693, 5912, and 4813, respectively. In all waves, the proportion of 18-29-year-olds responding to the surveys was higher than that of 30-44- or 50+-year-olds. Bangladesh (83.7% in Wave 1) and Uganda (70.1% in Wave 1) had a higher proportion of males than females, while it was different in Colombia (45.6% in Wave 1). Regarding the reporting of age and gender in survey waves, we observed a high agreement in all three countries; the Kappa statistic was 0.89 (agreement: 93.7%) from Wave 1 to Wave 2 and 0.90 (agreement: 94.5%) from Wave 1 to Wave 3. In Wave 1, the response and refusal rates were, respectively, 0.26% and 0.19% in Bangladesh; 0.65% and 0.89% in Colombia; and 2.63% and 0.71% in Uganda. From Wave 1 to Wave 2, the attrition rate was 37.2% in Bangladesh, 43.7% in Colombia, and 39.2% in Uganda. From Wave 1 to Wave 3, the attrition rate was 64.2%, 62.8%, and 58.4% in Bangladesh, Colombia, and Uganda, respectively. Despite high attrition across survey waves, the agreement about responses was substantial in all countries and MPS has the potential to be implemented in LMICs. More research is required to improve the retention and increase enrollment in some sociodemographic groups (e.g., older people or women). Future studies could also be benefitted from adding validation questions to ensure the participation by the same respondent.

**Supplementary Information:**

The online version contains supplementary material available at 10.1038/s41598-025-16809-5.

## Introduction

Rapid developments and advancements in mobile phone technology have taken place globally during the past two decades^1,2^. Few decades ago, the ownership and subscription rates of mobile phones were lower in low- and middle-income countries (LMICs) compared to high-income countries (HICs)^1^. However, these rates have increased during the recent years. For instance, in 2000, the subscription rate for mobile phones was 4 per 100 people in LMICs; this increased to 92 in 2015 and 104 in 2020. In contrast, in HICs, this rate was 48, 119, and 124 per 100 people in 2000, 2015, and 2020, respectively; the higher than 100% subscription rate is because of using multiple mobile phones by people^1,3^. Overall, in most LMICs, women are less likely to use and own mobile phones than men^4,5^.

The increasing subscription and ownership of mobile phones have created many opportunities to improve the health, education, or economic outcomes of nations. Many countries and organizations conduct mobile phone surveys (MPS) to collect health-related data^6–9^. This increased exponentially during the coronavirus disease 2019 (COVID-19) pandemic^10^. Furthermore, mobile phone surveys have some advantages over in-person surveys in collecting data from people living in remote and local areas of LMICs, which can significantly reduce the cost^11^. It can also be conducted conveniently and at a lower cost in some settings within a shorter period than in-person surveys^12^. However, MPS also has some limitations, including selection bias because of the difficulty of enrolling an adequate number of elderly or participants with lower education. Apart from that, because of the high volume of promotional or spam calls, people may feel reluctant to share their personal or health-related data via phone, especially related to culturally sensitive topics (e.g., contraceptive use)^13,14^.

Research has revealed ways to improve the performance of MPS^15^, and several data collection methods have been developed, including computer-assisted telephone interviews (CATI) and interactive voice response (IVR). In CATI, a human interviewer from a call center asks questions using a standard script and records the responses. IVR participants respond to preprogrammed audio interviews using their phone’s keypad (e.g., “Press 1 for Yes”)^16–18^. Studies reported a higher CATI response rate than IVR^17^. Despite the progress of MPS research methods, most previous studies were conducted with a single interview using a cross-sectional study design. MPS’s participation rates (e.g., response rate or cooperation rate) are substantially lower than in-person surveys. Although previous research has shown that MPS can collect longitudinal or panel data^19,20^, the panel was created using household sampling techniques where enrollment was initiated at the respondents’ households. It is unclear if a panel of survey respondents can be created and maintained in LMICs using random digit dialing (RDD) techniques. A significant benefit of using an RDD sampling approach is the savings in travel and personnel costs associated with in-person recruitment^21^. Participants’ retention or dropout rates across waves of MPS panels are not known when the sample is drawn without a known working sample frame.

In most LMICs, a sample frame of working phone numbers is difficult to obtain. Moreover, to make the sample nationally representative, a sampling frame of working phone numbers would be challenging to obtain from the whole country, primarily covering rural and urban regions of all divisions^21,22^. During an RDD survey, how the retention rates differ by sociodemographic groups is unknown. If a panel’s retention or dropout rates are known, then future research could also be conducted to examine the reliability (e.g., reproducibility) of the reported data or how the reported data could differ across waves of longitudinal surveys. Furthermore, longitudinal surveys could be conducted to examine changes in health behavior or impacts on a population.

In this study, we attempted to fill these gaps in knowledge and conducted an MPS panel survey in three LMICs: Bangladesh, Colombia, and Uganda. We assessed the feasibility of creating a panel of survey participants (i.e., based on age and gender), and characterized the panel’s performance over time. Specifically, to test if the same participant responded to all of the surveys, we examined the agreement of responses regarding age and gender across survey waves. The findings of this study would help design MPS to collect health-related data in LMICs.

## Methods

This study’s MPS was delivered in three waves among adults (i.e., at least 18 years old) in all three countries (i.e., Bangladesh, Colombia, and Uganda). These three LMICs are in South Asia, South America, and East Africa, respectively^23^. According to the latest estimates of the International Telecommunication Union, the mobile phone subscription rates in Bangladesh, Colombia, and Uganda were 1, 6, and 1 per 100 people in 2000 and 105, 156, and 70 per 100 people in 2022, respectively^3,24^. The proportions of rural population in Bangladesh, Colombia and Uganda were 62%, 19%, and 75% in 2020, respectively^25^. From Wave 1 to Wave 3, the same group of participants received the surveys. Participants were recruited from six age-gender strata (i.e., 18-29-, 30-44-, and 45+-year-old males and females). The surveys were sent at two-week intervals. Data collection took place between February and April 2023.

The first IVR surveys (wave 1) were sent to randomly generated mobile phone numbers between 8 am and 8 pm local time. Upon answering the phone, participants received instructions about the MPS, including information on the survey’s purpose, the expected time commitment, the survey’s sponsoring agency, and the requirements for an airtime incentive. Participants were assured that the shared information would be kept confidential and private; for most questions, instructions were given to press 1 for yes and 3 for no.

Then, participants were asked whether they were at least 18 years old; if the answer was ‘yes’ (i.e., by pressing 1), consent was obtained. Consenting participants were asked additional demographic questions (e.g., gender, location, or education). Then, tobacco use and other NCD-related questions were asked. They also had to respond to questions related to gender norms (a subtype of social norms related to gender differences). The NCD-related and gender norms questions were divided into three parts to be sent in three waves. The age and gender questions were the only common questions in all three surveys and in this manuscript, we only included findings related to agreement of reporting age-gender along with retention by age-gender in all 3 surveys. Lastly, participants were asked about their interest in taking a survey after two weeks; those who indicated yes were included in the panel. If a participant did not participate in wave 2, s/he did not receive wave 3. The average duration of complete surveys in wave 1, wave 2, and wave 3 was 6, 5, and 6 min, respectively.

IVR surveys were sent by an MPS provider, EngageSPARK, a company that specializes in delivering IVR and SMS surveys and deploying RDD approaches globally. EngageSPARK was subcontracted by investigators from Johns Hopkins University’s Bloomberg School of Public Health. Permissions for data processing were carried out according to the regulations of each country, where a local ethics committee reviewed and approved the protocol. A local ethics committee reviewed and approved the protocol from each country. All methods were performed in accordance with the relevant guidelines and regulations of each country.

### Sample size calculation

We targeted a sample size of 385 respondents per age-gender strata, which was calculated with the Wald statistics formula assuming an expected prevalence of 50% risk factor (*p* = 0.5), a 5% margin of error (δ = 0. 05), and a 5% type-I error (α = 0. 05). An assumption of 50% prevalence is conservatively preferred, which gives the largest sample size when exact prevalence is not known. Based on findings from a study in Honduras and Peru, we assumed an 80% attrition rate and targeted (385 × 6 age-gender strata)/(1-0.20) = 11,550 sample size. We were able to meet sample size requirements for younger people. However, we did not meet sample size requirements for 45+-year-old people due to the high cost of conducting each interview of that age group. We had to make a high number of phone calls to complete interviews in the older age groups, which resulted in higher costs for them.

### Statistical analysis

First, to provide survey disposition codes to each phone call and obtain participation rates (i.e., contact, response, refusal, and cooperation rates), we used the standard definitions and equations provided by the American Association for Public Opinion Research (AAPOR). The AAPOR equations and definitions are presented in Supplemental Table 1. Next, we reported the sociodemographic characteristics of the respondents. We also reported the attrition rates across waves by age and gender groups. As age and gender were asked in all three waves, we compared the ‘agreement’ of the responses across waves with Kappa statistics. We reported the Pearson correlation coefficient between the age (in year) responses across the waves as well. This study aimed to obtain an MPS panel that can be used to obtain longitudinal data; however, we were not sure whether the same participant took all the surveys and we did not collect identifiable data to ensure privacy of the respondents. Therefore, the Kappa statistics and correlation coefficient aimed to show whether the same participants received the surveys. The analyses were performed with Stata 15.0 (Stata Corporation, College Station, TX, USA).

## Results

Table 1 shows the response rate by survey wave. In Wave 1, the contact, response, refusal, and cooperation rates were, respectively, 0.45%, 0.27%, 0.18%, and 53.10% in Bangladesh; 1.49%, 0.63%, 0.86%, and 36.43% in Colombia; and 3.35%, 2.63%, 0.71%, and 63.91% in Uganda. All the rates were higher in Waves 2 and 3 than in Wave 1. For instance, in Wave 2, the response rate was 53.25% in Bangladesh, 59.70% in Colombia, and 70.97% in Uganda; in Wave 3, the rate was 62.02% in Bangladesh, 69.61% in Colombia, and 73.02% in Uganda.

**Table 1:** Disposition codes of the mobile phone calls and survey participant rates by country, February to April 2023.

Variable	Bangladesh			Colombia			Uganda		
	W1	W2	W3	W1	W2	W3	W1	W2	W3
Disposition codes, n^1^									
Complete (I)	2717	1696	964	5912	3331	2198	4813	2928	2004
Partial (P)	390	132	50	981	136	72	1121	153	58
Refusal (R)	769	23	14	8384	2035	841	1183	47	43
Breakoffs (R)	1241	127	39	952	120	104	414	368	98
Unknown (U)	1,128,424			1,075,531			217,343		
Non-contact (NC)		1455	568		185	46		845	621
Age ineligible	6098	280	61	604	105	70	792	472	104
Total	1,139,639	3713	1696	1,092,364	5912	3331	225,666	4813	2928
Participation rates, %^2^									
Contact	0.45	57.6	65.3	1.5	96.8	98.6	3.4	80.5	78.0
Response	0.27	53.3	62.0	0.6	59.7	69.6	2.6	71.0	73.0
Refusal	0.18	4.4	3.2	0.9	37.1	29.0	0.7	9.6	5.0
Cooperation	53.10	85.7	90.4	36.4	59.3	68.4	63.9	83.8	91.0

^1^Complete survey: Participants completed all survey modules; Incomplete: Did not complete all modules; Refusal: Did not provide consent; Breakoffs: Participant disconnected before any module completion; Unknown: Did not select age-gender; Non-contact: Did not pick up the phone.

^2^Contact rate: (I + P + R)/(I + P + R + U + NC); Response rate: (I + P)/ (I + P + R + U + NC); Refusal rate: R/(I + P + R + U + NC); Cooperation rate: (I))/(I + P + R).

Table 2 shows the sociodemographic characteristics by survey mode. The proportion of younger people (i.e., 18-29-year-olds) was higher in all three countries than older people (i.e., 30+-year-olds). Although the proportion of males was higher in Bangladesh and Uganda than their female counterparts; it was the opposite in Colombia. The proportion of rural residents was higher in all three countries than urban residents.

**Table 2:** Sociodemographic characteristics of the respondents by country from wave 1 to wave 3, February to April 2023, % (N).

Variable	Bangladesh			Colombia			Uganda		
	Wave 1	Wave 2	Wave 3	Wave 1	Wave 2	Wave 3	Wave 1	Wave 2	Wave 3
Age (years)									
18–29	65.6 (1778)	68.4 (1160)	69.7 (672)	43.7 (2585)	45.7 (1521)	45.8 (1006)	60.1 (2891)	60.5 (1772)	60.7 (1216)
30–44	28.0 (758)	27.1 (460)	27.1 (261)	34.9 (2062)	35.3 (1175)	35.8 (787)	31.4 (1513)	31.7 (928)	32.7 (655)
45+	6.4 (174)	4.5 (76)	3.2 (31)	21.4 (1265)	19.1 (635)	18.4 (405)	8.5 (409)	7.8 (228)	6.6 (133)
Gender									
Male	83.7 (2260)	84.9 (1436)	83.5 (805)	45.6 (2694)	42.7 (1421)	41.9 (920)	70.1 (3376)	71.4 (2090)	72.0 (1443)
Female	15.6 (420)	15.0 (253)	16.3 (157)	53.3 (3152)	56.6 (1887)	57.2 (1258)	27.5 (1324)	27.3 (799)	27.1 (544)
Other	0.7 (19)	0.2 (3)	0.2 (2)	1.1 (66)	0.7 (23)	0.9 (20)	2.3 (113)	1.3 (39)	0.8 (17)
Age & gender									
18–29 y, M	53.6 (1432)	56.8 (959)	57.0 (548)	20.4 (1190)	20.2 (667)	20.1 (437)	41.0 (1926)	42.2 (1219)	42.1 (837)
18–29 y, F	11.9 (318)	11.5 (195)	12.8 (123)	23.3 (1360)	25.5 (843)	25.7 (560)	19.2 (903)	18.2 (525)	18.5 (367)
30–44 y, M	25.0 (668)	24.1 (407)	23.9 (230)	15.7 (917)	14.2 (471)	14.3 (312)	24.5 (1153)	24.1 (697)	25.5 (507)
30–44 y, F	3.1 (84)	3.1 (52)	3.1 (30)	19.3 (1128)	21.0 (696)	21.4 (467)	6.9 (325)	7.8 (225)	7.2 (144)
45 + y, M	5.8 (154)	4.1 (70)	2.8 (27)	10.0 (587)	8.6 (283)	7.9 (171)	6.3 (297)	6.0 (174)	5.0 (99)
45 + y, F	0.7 (18)	0.4 (6)	0.4 (4)	11.4 (664)	10.5 (348)	10.6 (231)	2.0% (96)	1.7% (49)	1.7 (33)
Location^1^									
Urban	44.5 (1208)			27.4 (1619)			49.3 (2375)		
Rural	55.5 (1506)			72.6 (4293)			50.7 (2438)		
Education^1^									
No school	7.5 (204)			8.7 (513)			15.8 (761)		
Primary	17.0 (460)			16.0 (945)			26.3 (1265)		
SSC/O-level/high school^2^	33.3 (901)			36.2 (2139)			26.8 (1288)		
HSC/A-level/technical^3^	19.4 (524)			24.0 (1420)			11.2 (537)		
Graduation	22.8 (616)			15.1 (895)			20.0 (962)		

^1^Location and education data not collected in Waves 2 and 3.

^2^SSC/O-Level in Bangladesh, O-level in Uganda, and High school in Colombia.

^3^HSC/A-Level in Bangladesh, A-level in Uganda, and Technical school in Colombia.

*SSC* Secondary school certificate, *HSC* Higher secondary certificate.

As seen in Table 3, very high agreement (about 90%) was observed regarding age and gender among people who completed the interviews in all three countries. For instance, from Wave 1 to Wave 2, the Kappa statistic regarding age and gender was 0.86 (agreement: 91.5%) in Bangladesh, 0.90 (agreement: 91.6%) in Colombia, and 0.80 (agreement: 85.2%) in Uganda; from Wave 1 to Wave 3, this Kappa statistic was 0.87 (agreement: 92.5%) in Bangladesh, 0.90 (agreement: 91.6%) in Colombia, and 0.82 (agreement: 87.0%) in Uganda.

**Table 3:** Agreement of age-gender among participants of completed surveys by country, February to April 2023.

Variable	Bangladesh	Colombia	Uganda
Age (years)			
Kappa (W 1–2)	0.89 (Agreement: 95.0%, *p* < 0.001)	0.93 (Agreement: 95.5%, *p* < 0.001)	0.83 (Agreement: 91.1%, *p* < 0.001)
Kappa (W 2–3)	0.93 (Agreement: 96.8%, *p* < 0.001)	0.96 (Agreement: 97.3%, *p* < 0.001)	0.89 (Agreement: 94.0%, *p* < 0.001)
Kappa (W 1–3)	0.90 (Agreement: 95.4%, *p* < 0.001)	0.94 (Agreement: 96.1%, *p* < 0.001)	0.85 (Agreement: 92.0%, *p* < 0.001)
Gender			
Kappa (W 1–2)	0.82 (Agreement: 95.3%, *p* < 0.001)	0.89 (Agreement: 94.6%, *p* < 0.001)	0.75 (Agreement: 89.5%, *p* < 0.001)
Kappa (W 2–3)	0.89 (Agreement: 97.1%, *p* < 0.001)	0.89 (Agreement: 94.4%, *p* < 0.001)	0.82 (Agreement: 92.9%, *p* < 0.001)
Kappa (W 1–3)	0.83 (Agreement: 95.7%, *p* < 0.001)	0.86 (Agreement: 93.1%, *p* < 0.001)	0.79 (Agreement: 91.1%, *p* < 0.001)
Age & gender			
Kappa (W 1–2)	0.86 (Agreement: 91.5%, *p* < 0.001)	0.90 (Agreement: 91.6%, *p* < 0.001)	0.80 (Agreement: 85.2%, *p* < 0.001)
Kappa (W 2–3)	0.91 (Agreement: 94.9%, *p* < 0.001)	0.91 (Agreement: 93.0%, *p* < 0.001)	0.85 (Agreement: 89.1%, *p* < 0.001)
Kappa (W 1–3)	0.87 (Agreement: 92.5%, *p* < 0.001)	0.90 (Agreement: 91.6%, *p* < 0.001)	0.82 (Agreement: 87.0%, *p* < 0.001)

**Fig. 1:**
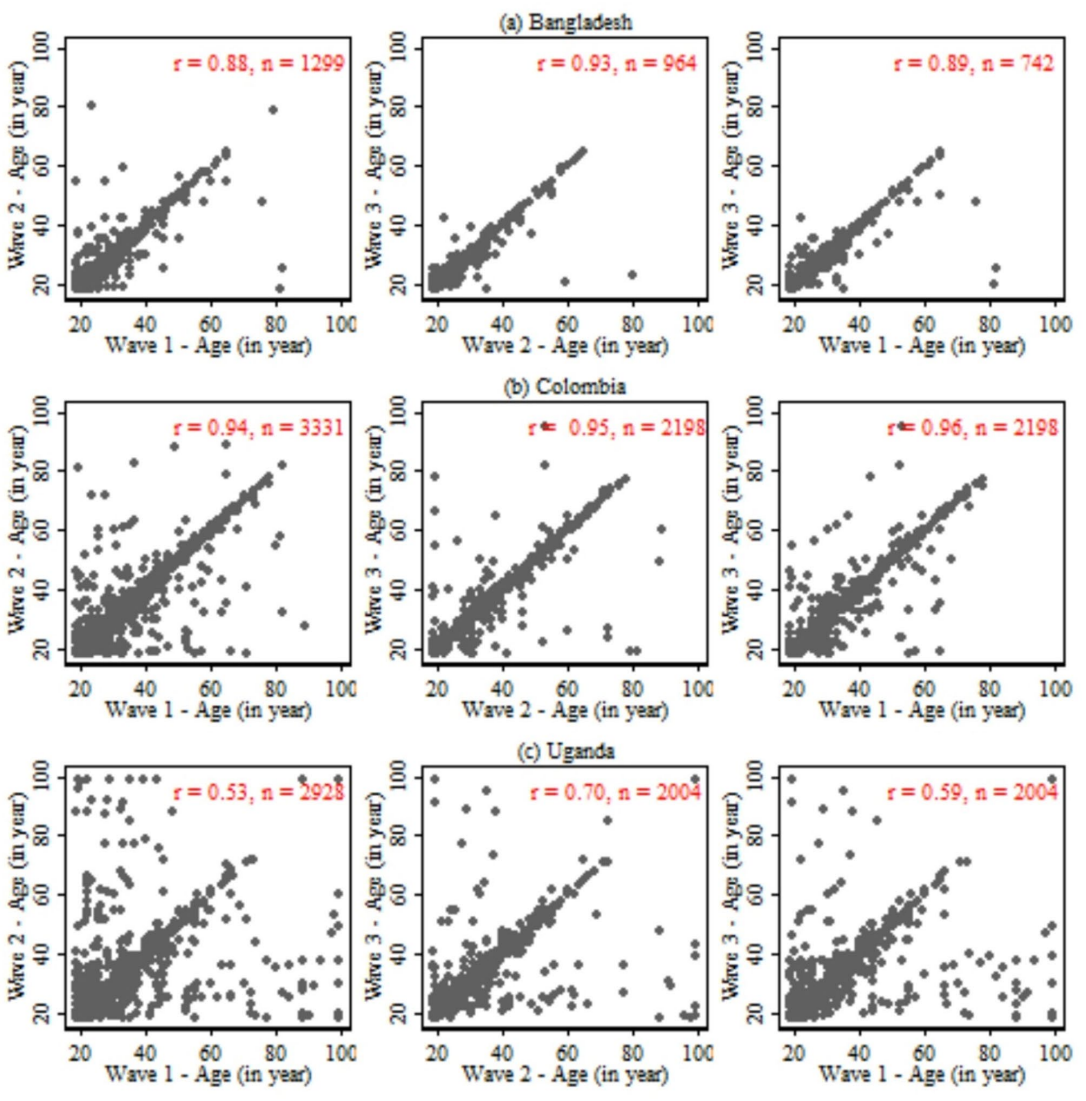
Pearson correlation coefficient (r) for the agreement of age (in year) responses across survey waves, February to April 2023.

In Fig. 1, when we looked into the correlation of age by survey waves in all three countries, similar to the Kappa statistic reported in Table 2, a higher correlation was observed in Bangladesh (e.g., 0.88 between Waves 1 and 2); and Colombia (e.g., 0.94 between Waves 1 and 2). However, the correlation was lower in Uganda (e.g., 0.53 between Waves 1 and 2).

Table 4 shows the attrition rate (%) across the completed surveys. From Wave 1 to Wave 2, the attrition rate was 37.2% in Bangladesh, 43.7% in Colombia, and 39.2% in Uganda; from Wave 1 to Wave 3, this rate was 64.2% in Bangladesh, 62.8% in Colombia, and 58.4% in Uganda. The attrition rate among older people was higher than that of their younger counterparts in all three countries. For instance, in Bangladesh, from Wave 1 to Wave 2, the attrition rate among 18-29-year-olds was 33.0% among males and 38.7% among females.

**Table 4:** Attrition rate (%) by self-reported age and gender across completed surveys in each country, February to April 2023.

Age (years)	Attrition, %					
	Wave 1 to 2 (w1 − w2)/w1		Wave 2 to 3 (w2 − w3)/w2		Wave 1 to 3 (w1 − w3)/w1	
	M	F	M	F	M	F
Bangladesh						
18–29	33.0	38.7	42.3	39.5	61.4	62.9
30–44	39.1	38.1	43.2	46.2	65.4	66.7
45+	54.6	66.7	55.7	50.0	79.9	83.3
18+	36.3	39.8	43.2	41.1	63.8	64.5
Total^1^	37.2		43.0		64.2	
Colombia						
18–29	43.9	38.0	35.4	32.6	63.8	58.2
30–44	48.6	38.3	36.1	31.0	67.2	57.4
45+	51.8	47.6	39.9	33.3	71.0	65.1
18+	47.3	40.1	36.5	32.2	66.5	59.4
Total^1^	43.7		34.0		62.8	
Uganda						
18–29	36.7	41.9	31.3	30.1	56.5	59.4
30–44	39.5	30.8	27.3	36.0	56.0	55.7
45+	41.4	49.0	43.1	32.7	66.7	65.6
18+	38.1	39.7	28.4	31.9	57.3	58.9
Total^1^	39.2		31.6		58.4	

^1^Does not include people who answered “Other”. See supplemental table for additional data.

*M* Male, *F* Female, *W* Wave.

## Discussion

Although we observed a moderately high attrition rate across survey waves in this study, the overall agreement between age and gender responses was substantial. The attrition across surveys also differed by sociodemographic characteristics, including age and gender. When we looked into the agreement, responses were similar to those sociodemographic characteristics. Overall, we were able to obtain a large sample within a short period of time in these countries. This study adds to the growing body of literature studying MPS’s usefulness in collecting population-based data.

Despite survey panel creation by previous studies, to our knowledge, this is the first study to report feasibility, attrition, and agreement using RDD across three waves with one cohort in three LMICs. Knowing the participation, retention, or dropout rates of a panel would enable the possibility of further studies. Such studies could investigate the reliability of the data (e.g., reproducibility) or explore variations in the data over successive rounds of longitudinal surveys. For instance, Dillon conducted a panel study in rural Tanzania; this study had several waves, and the survey had a lower attrition rate than ours^19^. However, the survey sample was not obtained through RDD, and they provided mobile phones to all participants. Another study by the World Bank, as a part of the projects “Listening to Africa (L2A)”, “Listening to Latin America (L2LA)”, and “Listening to the Caribbean (L2C)”, used sampling from household phone numbers as well^26^. The primary purpose was to use MPS as a complement to ongoing in-person surveys, not to study MPS’s feasibility in collecting random population-level data^19,25^.

Although this study had low participation and high attrition rates, we observed a significant agreement among participants across waves. The low retention or high attrition in some sociodemographic groups (e.g., older people) indicates that future studies have to enroll a higher number of participants from those groups in wave 1 to have their higher number in wave 3. The lack of agreement regarding age and gender in some cases could result from the fact that a phone is shared by multiple household members, and during the second and third interviews, different household members picked up the call^16,19,27^. However, the overall high level of agreement indicates that MPS using IVR can obtain reliable estimates across waves similar to in-person waves. This may be useful if a panel is responding to data collection requests on topics that require a long time to interview or to collect data on responses that require follow-up to understand the consequences (e.g., the consequences of COVID-19)^20^. Future studies could also be benefitted from adding validation questions to ensure the participation by the same respondent.

Similar to previous studies, the survey’s overall participation (i.e., response or cooperation rate) in all three countries was substantially lower during wave 1^15,28,29^. Moreover, during wave 1, the number of people from some sociodemographic groups, especially those of older age, was substantially lower, and we were not able to obtain a sufficient sample size for them. We could not get adequate females in Bangladesh and Uganda, which was the opposite in Colombia. Without improving participation among all sociodemographic groups, the findings of an MPS cannot be generalizable. Gender contexts vary among the three nations, which is reflected in cell phone availability (e.g., Bangladesh’s 2021 gender development score was 0.898, Colombia’s 0.984, and Uganda’s 0.927)^30^. Without improving participation among all sociodemographic groups, the findings of an MPS cannot be generalizable. In most LMICs, mobile phone ownership is lower among females or older people than their counterparts, which may explain their low participation^31^. The proportion of older people is also low in LMICs. Women also tend to spend more time doing household chores or taking care of children in these countries, which may make them less available for such phone surveys^32,33^. The privacy issue might also be another reason for the low participation; women may be unable to be in a private location to answer questions, or if they are with others within the household^13,14^. We provided an airtime incentive to improve survey participation, but that was insufficient. Previous studies employed inverse proportional weighting to generate sample weights from known population margins^33^. A sufficient sample size is required in all age, gender, education, or location strata to create the weight. We did not have enough sample sizes in all survey waves to apply that. Future studies should explore ways to improve participation among these underrepresented population groups.

It is also important to note that using different formulas or denominators may result in other estimates of response rates. Our method of calculating the response rate was conservative. We labeled all the phone calls as ‘unknown’ when a participant did not pick them up. Therefore, a certain proportion of these phone numbers could be inactive, and we counted them in the denominator when calculating the response rate. When we calculated the cooperation rate, we counted all the people who picked up the phone calls in the denominator, which resulted in a high cooperation rate, with the numerator being complete interviews^15,17^. A working sample frame of all active numbers may have improved the response rate. A working frame would reduce the cost of conducting phone calls as well.

We did not compare the costs of surveying by conducting parallel in-person surveys. In a previous study, we observed that the average cost of IVR was lower than household surveys to recruit younger people; however, it was significantly higher to recruit older people^33^. More research is required to reduce IVR costs, primarily to recruit older people or females.

This study has several strengths. First, this sample included all mobile phone operators in each country, which removed any potential selection bias resulting from the differences in subscribers’ characteristics among mobile operators. The data were collected anonymously; therefore, the risk of social desirability bias was minimized.

This study has some limitations as well. Although we obtained a large sample by RDD, our sample size was substantially low in some age-gender strata. The study was designed to create an NCD panel; the information from the panel would be based on self-reports; therefore, this information (e.g., information on physical activity) would be subject to recall bias. IVR would only include people with mobile phones; the information regarding NCD from people without mobile phones would remain unknown. Our follow-up period was short (i.e., two weeks), and many studies would require a longer follow-up. Therefore, this study does not allow us to assess whether or not subjects are retained in the long term. The low number of people in some sociodemographic groups was also a major limitation of our study. As we used IVR method instead of CATI, that might have produced a little lower response rate. Lastly, except for Kappa statistics and Pearson’s correlation coefficient, we did not use any other method to ensure that the same participants were answering all three surveys; this may have caused some errors too, as we did not use other method of identifying.

## Conclusion

Although we were able to create a large panel of mobile phone users, validated by agreement in age and gender, in a short period in three LMICs, potential challenges remain regarding obtaining an adequate sample from some age-gender strata and minimizing attrition of participants across survey waves. Despite these challenges, future studies should be able to successfully create panels using validated methods to ensure that the same participants take all the surveys.

## Supplementary Information

Below is the link to the electronic supplementary material.

Supplementary Material 1

## Data Availability

Data will be available upon acceptance from the OpenICPSR repository database (https://www.openicpsr.org/openicpsr/.

## References

[1] International Telecommunication Union. Mobile cellular subscriptions (per 100 people). (2022). Available: https://data.worldbank.org/indicator/IT.CEL.SETS.P2

[2] Silver, L. Smartphone Ownership Is Growing Rapidly Around the World, but Not Always Equally. In: Pew Research Center [Internet]. 5 Feb 2019 [cited 22 Sep 2022]. Available: https://www.pewresearch.org/global/2019/02/05/smartphone-ownership-is-growing-rapidly-around-the-world-but-not-always-equally/

[3] The World Bank. Mobile cellular subscriptions (per 100 people). 29 Aug 2023 [cited 29 Aug 2023]. Available: https://data.worldbank.org/indicator/IT.CEL.SETS.P2

[4] GSMA. The Mobile Gender Gap Report 2020. (2020). Available: https://www.gsma.com/mobilefordevelopment/wp-content/uploads/2020/05/GSMA-The-Mobile-Gender-Gap-Report-2020.pdf

[5] GSMA. Bridging the gender gap: Mobile access and usage in low and middle-income countries. (2015). Available: https://www.gsma.com/mobilefordevelopment/wp-content/uploads/2016/02/Connected-Women-Gender-Gap.pdf

[6] Deloitte, L. L. P. & Report for the GSM Association. What is the impact of mobile telephony on economic growth? A. (2022). Nov Available: https://www.gsma.com/publicpolicy/wp-content/uploads/2012/11/gsma-deloitte-impact-mobile-telephony-economic-growth.pdf

[7] Harvey, E. J. mHealth and the change it represents. *Can. J. Surg. J. Can. Chir.* **62**, 148. 10.1503/cjs.007919 (2019). 10.1503/cjs.007919 PMC6738501 31134781

[8] Lum, T. Mobile goes global: The effect of cell phones on economic growth and development. (2011). Available: https://digitalcommons.bucknell.edu/cgi/viewcontent.cgi?article=1003&context=honors_theses

[9] McCool, J., Dobson, R., Whittaker, R. & Paton, C. Mobile health (mHealth) in Low- and Middle-Income countries. *Annu. Rev. Public. Health*. **43**, 525–539. 10.1146/annurev-publhealth-052620-093850 (2022). 34648368 10.1146/annurev-publhealth-052620-093850

[10] Institute of Epidemiology Disease Control and Research. National Bulletin of Public Health. Available:. (2023). https://www.iedcr.gov.bd/site/page/cfab0203-6009-4e98-8bd4-bc74cc4032b5/-.

[11] Leo, B., Morello, R., Mellon, J., Piexoto, T. & Davenport, S. Do Mobile Surveys Work in Poor Countries. (2012). Available: http://catalog.ihsn.org/index.php/catalog/4775/related_materials

[12] Okeleke, K. Achieving mobile-enabled digital inclusion in Bangladesh. (2021). Available: https://www.gsma.com/mobilefordevelopment/wp-content/uploads/2021/03/Achieving-mobile-enabled-digital-inclusion-in-Bangladesh.pdf

[13] Ali, J. et al. Ethics considerations in global mobile Phone-Based surveys of noncommunicable diseases: A conceptual exploration. *J. Med. Internet Res.* **19**, e110. 10.2196/jmir.7326 (2017). 28476723 10.2196/jmir.7326 PMC5438462

[14] Brown, W. et al. Challenges and solutions implementing an SMS text message-based survey CASI and adherence reminders in an international biomedical HIV PrEP study (MTN 017). *J. Biomed. Inf.* **80**, 78–86. 10.1016/j.jbi.2018.02.018 (2018). 10.1016/j.jbi.2018.02.018 PMC5920551 29501908

[15] Gibson, D. G. et al. Effect of airtime incentives on response and Cooperation rates in non-communicable disease interactive voice response surveys: randomised controlled trials in Bangladesh and Uganda. *BMJ Glob Health*. **4**, e001604. 10.1136/bmjgh-2019-001604 (2019). 31565406 10.1136/bmjgh-2019-001604 PMC6747927

[16] Gibson, D. G. et al. Evaluation of mechanisms to improve performance of mobile phone surveys in Low- and Middle-Income countries: research protocol. *JMIR Res. Protoc.* **6**, e81. 10.2196/resprot.7534 (2017). 28476729 10.2196/resprot.7534 PMC5438454

[17] Gibson, D. G. et al. Mobile phone surveys for collecting Population-Level estimates in Low- and Middle-Income countries: A literature review. *J. Med. Internet Res.* **19**, e139. 10.2196/jmir.7428 (2017). 28476725 10.2196/jmir.7428 PMC5438460

[18] Hyder, A. A. et al. Noncommunicable disease risk factors and mobile phones: A proposed research agenda. *J. Med. Internet Res.* **19**, e133. 10.2196/jmir.7246 (2017). 28476722 10.2196/jmir.7246 PMC5438453

[19] Dillon, B. Using mobile phones to collect panel data in developing countries. *J. Int. Dev.* **24**, 518–527. 10.1002/jid.1771 (2012).

[20] Nikoloski, Z. et al. Modelling COVID-19 vaccination status and adherence to public health and social measures, members of Eastern mediterranean region and Algeria. *Bull. World Health Organ.* **101**, 111–120. 10.2471/BLT.22.288655 (2023). 36733625 10.2471/BLT.22.288655 PMC9874377

[21] Research, D. Random Digit Dialling (RDD). In: DJS Research [Internet]. 30 Aug 2023 [cited 30 Aug 2022]. Available: https://www.djsresearch.co.uk/glossary/item/Random-Digit-Dialling-RDD

[22] Elliott, R. What is Random Digit Dialing? 29 Sep 2020 [cited 30 Aug 2022]. Available: https://www.geopoll.com/blog/what-is-random-digit-dialing/

[23] World Bank. World Bank Country and Lending Groups. [cited 31 Aug 2023]. (2023). Available: https://datahelpdesk.worldbank.org/knowledgebase/articles/906519-world-bank-country-and-lending-groups

[24] The World Bank. Mobile cellular subscriptions (per 100 people) - Bangladesh. (2020). Available: https://data.worldbank.org/indicator/IT.CEL.SETS.P2?locations=BD

[25] Gallup & The World Bank Listening to LAC (L2L) Pilot Final Report. (2017). Jun Available: https://www.worldbank.org/en/programs/listening-to-africa

[26] Gibson, D. G., Farrenkopf, B. A., Pereira, A., Labrique, A. B. & Pariyo, G. W. The development of an interactive voice response survey for noncommunicable disease risk factor estimation: technical assessment and cognitive testing. *J. Med. Internet Res.* **19**, e112. 10.2196/jmir.7340 (2017). 28476724 10.2196/jmir.7340 PMC5438455

[27] Labrique, A. et al. Improving success of non-communicable diseases mobile phone surveys: Results of two randomized trials testing interviewer gender and message valence in Bangladesh and Uganda. Pry JM, editor. PLOS ONE. ;18: e0285155. (2023). 10.1371/journal.pone.0285155 10.1371/journal.pone.0285155 PMC10208499 37224125

[28] Ali, J. et al. Remote consent approaches for mobile phone surveys of non-communicable disease risk factors in Colombia and uganda: A randomized study. Rashid TA, editor. *PLOS ONE*. **17**, e0279236. 10.1371/journal.pone.0279236 (2022). 36542631 10.1371/journal.pone.0279236 PMC9770397

[29] Kibria, G. M. A. & Nayeem, J. Trends and factors associated with mobile phone ownership among women of reproductive age in Bangladesh. Malta M, editor. PLOS Glob Public Health. ;3: e0001889. (2023). 10.1371/journal.pgph.0001889 10.1371/journal.pgph.0001889 PMC10198550 37205644

[30] L’Engle, K. et al. Survey research with a random digit dial National mobile phone sample in ghana: methods and sample quality. Ivers LC. *Editor PLOS ONE*. **13**, e0190902. 10.1371/journal.pone.0190902 (2018). 10.1371/journal.pone.0190902 PMC5774708 29351349

[31] Kibria, G. M. A. et al. Developing digital tools for health surveys in low- and middle-income countries: Comparing findings of two mobile phone surveys with a nationally representative in-person survey in Bangladesh. Kajungu D, editor. PLOS Glob Public Health. ;3: e0002053. (2023). 10.1371/journal.pgph.0002053 10.1371/journal.pgph.0002053 PMC10374008 37498841

[32] Deming, W. E. & Stephan, F. F. On a least squares adjustment of a sampled frequency table when the expected marginal totals are known. *Ann. Math. Stat.* **11**, 427–444. 10.1214/aoms/1177731829 (1940).

[33] The American Association for Public Opinion Research. Standard Definitions: Final dispositions of case codes and outcome rates for surveys. 9th ed. (2016).

