# Supplementary material for: A study in Bangladesh, Colombia, and Uganda on creating and retaining mobile health survey panels for longitudinal data collection
Source: Sci Rep. 2025 Sep 25;15:32875. doi: 10.1038/s41598-025-16809-5 (PMC12464302; doi:10.1038/s41598-025-16809-5)
Supplement: Supplementary file 1 — Supplementary Material 1 [file 41598_2025_16809_MOESM1_ESM.docx]

**Supplemental Table 1: American Association of Public Opinion Research (AAPOR) Definitions and Equations**

| **Disposition Codes** | | **Definition/Equations** |
| --- | --- | --- |
| **AAPOR Definitions** | Complete Interviews (I) | Participants who answered at least 5 of the 7 modules. |
|  | Partial Interviews (P) | Participants who answered 2,3, or 4 modules. |
|  | Refusals (R) | Participants who either did not press a button on their mobile phone to indicate consent, refused consent, or who hung-up at the consent question. |
|  | Break-offs (R) | Participants who consented but did not complete a module other than demographics |
|  | Unknown (U) | Participants who selected a survey language but did not answer the age question. |
|  | Estimated Unknown (eU) | Estimated proportion of unknown cases that were age eligible. |
|  | Ineligible on age | Participant who indicated an age less than 18 years of age. |
| **AAPOR Equations** | Contact Rate #2 | $\frac{(I+P+R+O)}{I+P+R+O+e(UH+UO)}$ |
|  | Response Rate #4 | $\frac{(I+P)}{I+P+R+O+e(UH+UO)}$ |
|  | Refusal Rate #2 | $\frac{(R)}{I+P+R+O+e(UH+UO)}$ |
|  | Cooperation Rate #1 | $\frac{(I)}{I+P+R+O}$ |
